# Effects of Different Types of Physical Activity on Respiratory Health Parameters in Elderly Males: A Cross-Sectional Study

**DOI:** 10.7759/cureus.59759

**Published:** 2024-05-06

**Authors:** Jitendra Bamrotia, Ashutosh N Joshi, Swapnil Paralikar, Rajesh Kathrotia, Vikas Kumar Patel, Rajathi Rajendran

**Affiliations:** 1 Statistics, Sir Sayajirao General Hospital, Vadodara, IND; 2 Physiology, Baroda Medical College, Vadodara, IND; 3 Physiology, Government Medical College, Bhavnagar, IND; 4 Physiology, All India Institute of Medical Sciences, Rajkot, IND; 5 Physiology, All India Institute of Medical Sciences, Bathinda, IND

**Keywords:** lung function, spirometry, respiratory health, physical activity, elderly

## Abstract

Background

*Population aging* is a global phenomenon associated with declines in muscle mass, physical activity levels, and respiratory health among elderly individuals. Despite evidence suggesting the benefits of physical activity on respiratory function, there is limited research examining its effects on lung function in the Indian elderly population.

Materials and methods

This cross-sectional study aimed to investigate the impact of different intensities of physical activity on respiratory health parameters among apparently healthy elderly males aged 60-80 years. Participants were categorized into walking, swimming, and sedentary groups based on their level of physical activity. Anthropometric measurements, cardiovascular parameters, respiratory endurance tests, and spirometry were conducted to assess lung function. Statistical analysis included nonparametric tests to compare the groups.

Results

Age, height, weight, BMI, waist circumference, and hip circumference were similar across groups, but the waist-to-hip ratio was higher in the sedentary group. Systolic and diastolic blood pressure did not differ significantly, while the pulse rate was lower in the swimming group. The sedentary group exhibited lower respiratory endurance, with a significantly reduced 40 mmHg endurance test and maximum expiratory pressure compared to the walking and swimming groups. Spirometry results showed significant improvements in various parameters, including forced vital capacity (FVC), forced expiratory volume in the first second (FEV1), peak expiratory flow rate (PEFR), forced expiratory flow 25% (FEF-25), and maximum voluntary ventilation (MVV) in the walking and swimming groups compared to the sedentary group.

Conclusion

Regular physical activity, particularly walking and swimming, appears to positively influence respiratory health parameters among elderly males. Engaging in these activities may enhance respiratory muscle strength and lung function, potentially mitigating age-related declines in pulmonary function and promoting overall well-being.

## Introduction

The global phenomenon of population aging presents a pressing challenge across nations. Projections for the European Union alone indicate a substantial increase in the population aged over sixty-five, from 85 million in 2008 to an estimated 151 million by 2060 [1]. Similarly, on a global scale, the population of individuals over the age of 65 is forecasted to surge from 461 million in 2004 to a staggering 2 billion by 2050 [2]. Such demographic shifts have profound implications for healthcare and social support systems, necessitating reevaluating how we address the needs of aging populations [3,4].

With advancing age, there is a notable decline in muscle mass and strength, contributing to a progressive reduction in physical activity levels. Studies suggest that physical activity often diminishes by 40%-80% as individuals age, heightening the risk of metabolic disorders and chronic diseases such as cancer, diabetes, and cardiovascular conditions. The aging process involves intricate changes across all organ systems, including alterations in lung function parameters, such as forced expiratory volume in one second (FEV1), which tends to decrease due to aging-related factors [3,4].

Research conducted in various countries, including the USA, Brazil, and China, has documented the decline in respiratory muscle strength with age among individuals from diverse ethnic backgrounds [5,6]. Additionally, evidence indicates that several physical therapy interventions can mitigate the adverse effects of aging on overall well-being [7]. Notably, leisure walking has been highlighted as a cost-effective form of exercise, requiring no specialized equipment or expertise [8].

Despite these insights, there remains a notable gap in our understanding of how disparities in physical activity levels impact lung function among older adults within the Indian population. This study aims to address this gap by investigating how varying intensities of physical activity influence lung function in older adults. By shedding light on this relationship, the findings of this study can inform targeted interventions to promote respiratory health among aging individuals in India.

## Materials and methods

This cross-sectional study was conducted at the Department of Physiology, Baroda Medical College, Gujarat. The study focused on apparently healthy males aged 60-80 years, drawn from nearby elderly care facilities in Vadodara, Gujarat. The inclusion criteria specified that participants had to be non-smokers with no history of respiratory conditions such as asthma or emphysema. The study's total number of participants (n) was 73, all providing written informed consent before inclusion.

Participants were categorized into three groups based on their level of physical activity as self-reported by the participants: the walking group, the swimming group, and the sedentary/control group. The walking group comprised individuals who engaged in regular walking for a minimum of 150 minutes per week, while the swimming group consisted of those who regularly swim for the same duration. The sedentary/control group included inactive individuals who did not meet the minimum physical activity criteria. A sedentary lifestyle was defined according to the Centers for Disease Control and Prevention criteria, which denotes a lack of leisure-time physical activity or engagement in activities lasting less than 20 minutes, fewer than three times per week [9].

All assessments were conducted at the institute between 9 am and 12 pm. Participants underwent evaluations of overall health and physical activity, as well as measurements of anthropometric parameters. Additionally, a thorough systemic examination was performed. The same investigator conducted all recordings to minimize instrumental and observer bias, and the instruments used were regularly calibrated.

This study design aimed to investigate the potential effects of different types of physical activity on the health parameters of elderly males, with a focus on respiratory health and overall well-being. The anthropometric measurements in the study were conducted as described (Table 1).

**Table 1:** Details of Anthropometric measurements in the study.

Sr. No.	Anthropometric parameter	Description of measurement
1	Weight	Weight in kilograms (kg) was measured using a weighing machine (Omron HN 289, Japan) with participants wearing minimal clothing.
2	Height	Height in centimeters (cm) was measured using a standalone stadiometer (Prime Surgicals, India).
3	Body Mass Index (BMI)	BMI was calculated using the Quetlets formula, which involves dividing weight in kilograms by the square of height in meters (kg/m²).
4	Waist Circumference	Waist circumference was measured using an anthropometric tape (Cescorf, Brazil). The measurement was taken at the midpoint between the costal margin and iliac crease in the mid-axillary line.
5	Hip Circumference	Hip circumference was measured in centimeters using the same measuring tape. The measurement was taken at the widest portion of the buttocks, with the tape parallel to the floor. Both waist and hip circumference measurements were taken with participants standing, feet closer together, arms crossing in front over the shoulder, body weight evenly distributed, and wearing minimal clothing. Additionally, the measurements were taken at the end of a normal expiration.
6	Waist-to-Hip Ratio (WHR)	WHR was calculated using the measurements of waist circumference and hip circumference. WHR is obtained by dividing the waist circumference by the hip circumference.

The study conducted spirometry to assess various respiratory parameters using a MEDI: SPIRO digital spirometry system and software (Maestros Mediline Systems Ltd., Navi Mumbai, India). The details of respiratory parameters measurement are described (Table 2)

**Table 2:** Details of respiratory parameter measurement using Spirometer.

Sr. No.	Respiratory parameter	Description of measurement using spirometer
1	Forced Vital Capacity (FVC)	FVC represents the total amount of air that a person can exhale forcefully after taking a deep breath. It is measured in liters (L) or milliliters (mL).
2	Forced Expiratory Volume in 1 Second (FEV1)	FEV1 measures the volume of air forcibly exhaled in the first second of a forced expiration. It is also measured in liters (L) or milliliters (mL).
3	Peak Expiratory Flow (PEF)	PEF measures the maximum speed at which air is exhaled during a forced expiration. It is typically measured in liters per minute (L/min) or liters per second (L/s).
4	Maximal Voluntary Ventilation (MVV)	MVV represents the maximum volume of air that a person can inhale and exhale in one minute during rapid, deep breathing. It is often expressed in liters per minute (L/min).

Spirometry techniques were performed following the norms set by the Thoracic Society of Australia and New Zealand, ensuring standardized procedures and reliable measurements [10]. Three technically acceptable trials were conducted for each parameter, and the highest score within a ±5% variance was included for analysis to maintain accuracy and consistency. Participants were given a two-minute rest period between trials to minimize fatigue and ensure reliable results.

40 mmHg Endurance Test and Maximal Expiratory Pressure Test (MEPT)

A mercury manometer, devoid of a pressure cuff, served as the primary apparatus for conducting both the 40 mmHg endurance test and the maximal expiratory pressure test (MEPT). The procedure involved the following steps:

Participants were seated comfortably on a stool, ensuring a relaxed posture. A nasal clip was applied to the participant to prevent nasal airflow interference. Participants were instructed to inhale deeply, followed by exhaling consistently into the mercury manometer through a rubber tube, aiming to maintain a steady pressure of 40 mmHg. The duration for which the individual could sustain the mercury column at 40 mmHg was meticulously recorded in seconds. If the participant failed to achieve the target pressure of 40 mmHg, the recorded duration was 0 seconds. Following the initial test, participants were allowed a rest period of 2 minutes to minimize potential fatigue effects.

Subsequently, participants were again instructed to inhale deeply and exhale into the mercury manometer tube to elevate the mercury column to its maximum height. The maximum level attained by the mercury column during this phase was measured in millimeters of mercury (mmHg). This value denoted the Maximum Expiratory Pressure (MEP) and indicated the participant's respiratory muscle strength under maximal exertion.

Statistical analysis

The data obtained from all participant groups underwent statistical analysis utilizing SPSS version 19 software (IBM Corporation, Armonk, NY, USA). The normal distribution of the data was assessed using the Shapiro-Wilk test. Due to the non-normal distribution observed, nonparametric tests, particularly the Kruskal-Wallis test with post hoc analysis, were applied to compare the three groups.

A predetermined significance level of p<0.05 was established to indicate statistical significance in all analyses. Results are presented as median values with interquartile ranges (IQR) where appropriate.

## Results

Out of a total of 73 participants in the study, 31 were sedentary, 25 were in the walking group, and 17 were in the swimming group. Baseline parameters are compared among the three groups (Table 3). Age, height, weight, BMI, waist circumference, and hip circumference were found to be similar across all groups. However, the waist-to-hip ratio was notably higher in the sedentary group compared to both the walking and swimming groups. Systolic and diastolic blood pressure did not exhibit significant differences among the groups, while the pulse rate was observed to be lower in the swimming group compared to both the walking and sedentary groups.

**Table 3:** Comparison of anthropometric and cardiovascular baseline parameters across the groups Comparison across groups was done using the Kruskal Wallis test, and post hoc pairwise comparisons were done. *p<0.05 in comparison with the sedentary group. #p<0.05 on comparison with a walking group BMI: body mass index; SBP: Systolic blood pressure; DBP: Diastolic blood pressure.

Parameters	Sedentary (n=31)	Walking (n=25)	Swimming (n=17)	Kruskal Wallis (p-value)
	Mean ±SD	Mean ±SD	Mean ±SD	
Age (years)	70.94 ±5.3	69.48 ± 5.23	67.00 ± 5.10	0.060
Height (cm)	161.61± 7.55	164.08 ± 7.64	165.76 ± 7.04	0.180
Weight (kg)	66.32 ± 16.03	69.20 ± 11.70	71.88 ± 13.02	0.070
BMI (kg/m^2^)	25.33 ± 5.62	25.72 ±4.17	26.19 ± 4.19	0.220
Waist Circumference (cm)	98.97 ± 13.79	95.68 ±10.26	98.29 ± 11.29	0.680
Hip circumference (cm)	96.58 ± 8.88	97.80 ± 7.74	100.94 ± 8.39	0.120
Waist/Hip (W/H) ratio	1.02 ± 0.07	0.98* ± 0.05	0.98* ± 0.06	0.020
Pulse (beats per min)	76.13 ± 11.12	72.00 ± 13.43	69.41*^#^ ± 5.47	0.040
SBP (mmHg)	131.87 ±18.42	132.64 ± 15.73	136.12 ± 9.31	0.710
DBP (mmHg)	77.16 ± 8.91	76.40 ± 9.26	79.20 ± 7.04	0.063

The 40 mmHg endurance test and maximum expiratory pressure were significantly lower in the sedentary group when compared to both the walking and swimming groups, while breath-hold time did not exhibit significant differences among the groups. The respiratory endurance parameters are displayed (Table 4)

**Table 4:** Comparison of respiratory endurance parameters across the groups Comparison across groups was done using the Kruskal Wallis test, and post hoc pairwise comparisons were done. *p <0.05 in comparison with the sedentary group.

Parameter	Sedentary (n=31)	Walking (n=25)	Swimming (n=17)	Kruskal Wallis (p-value)
	Mean ± SD	Mean ± SD	Mean ± SD	
Breath-hold time (s)	24.33 ± 9.02	25.68 ± 9.30	30.59 ± 10.20	0.070
40mm Endurance test (s)	16.37 ±13.60	22.68* ± 7.24	25.29* ± 12.02	0.020
Maximum expiratory pressure (mmHg)	57.43 ±24.65	80.36* ±21.66	93.24* ±22.57	0.001

Spirometry results, as illustrated (Table 5), revealed that forced vital capacity (FVC) was significantly higher in the swimming group compared to both the sedentary and walking groups. Forced expiratory volume in the first second (FEV1), peak expiratory flow rate (PEFR), forced expiratory flow 25% (FEF-25), and maximum voluntary ventilation (MVV) demonstrated significant improvements in both the walking and swimming groups in comparison to the sedentary group. Other respiratory function parameters remained comparable across all groups.

**Table 5:** Comparison of pulmonary function parameters across the groups Comparison across groups was done using the Kruskal Wallis test, and post-hoc pairwise comparisons were done. *p <0.05 in comparison with the sedentary group. # p <0.05 on comparison with a walking group. FVC(L): Force Vital Capacity (Litres); FEV-05(L): Force Vital Capacity 05 (Litres); FEV1(L): Force Vital Capacity in 1 second (Litres); FEV 3(L): Force Vital Capacity in 3 sec (Litres); FEV1/FVC (%): Force Vital Capacity in 1 Second/Force Vital Capacity;  FEV-3/FVC (%): Force Vital Capacity in 3 Seconds/Force Vital Capacity;  FEF25-75(L): Force expiratory flow 25-75 (Litres); PEF (L/sec): Peak expiratory flow (Litres/Seconds); FEF-25(L): Force expiratory flow 25 (Litres); FEF-50(L): Force expiratory flow 50 (Litres); FEF-75(L): Force expiratory flow 75 (Litres); MVV (L/min): Maximum voluntary ventilation liters/sec.

Parameter	Sedentary (n=31)	Walking (n=25)	Swimming (n=17)	Kruskal Wallis ( p-value)
	Mean ±SD	Mean ±SD	Mean ±SD	
FVC (L)	2.00 ± 0.81	2.53 ± 1.29	2.67*^#^ ± 0.49	0.019
FEV-05 (L)	0.66 ± 0.42	1.11* ± 0.60	1.23* ± 0.54	0.001
FEV1 (L)	1.26 ± 0.54	1.76* ± 0.81	1.84* ± 0.44	0.001
FEV3 (L)	1.95 ± 0.51	2.36 ± 1.29	2.27 ± 0.44	0.056
FEV1/FVC (%)	66.26 ±17.90	70.77 ± 13.81	69.04 ± 11.12	0.500
FEV-3/FVC (%)	86.49 ± 8.55	86.76 ± 8.53	86.11 ± 7.39	0.621
FEF25-75 (L)	1.17 ± 0.78	1.69 ± 1.24	1.53 ± 1.22	0.219
PEF (L/sec)	3.35 ± 1.27	5.35* ± 1.88	5.77* ± 1.25	0.001
FEF-25 (L)	2.71 ± 1.23	4.42* ±2.11	4.80* ± 1.66	0.001
FEF-50 (L)	1.60 ± 1.03	2.38 ± 1.40	2.22 ± 1.38	0.058
FEF-75 (L)	0.53 ± 0.49	0.66 ± 0.69	0.55 ± 0.62	0.623
MVV (L/min)	50.37 ± 28.64	82.52* ±35.45	100.24 ± 29.10	0.001

## Discussion

The present study aimed to investigate the impact of different types of physical activity on respiratory health parameters among elderly males. Our findings reveal several notable observations regarding anthropometric measurements, respiratory endurance, and spirometry results across the studied groups.

Anthropometric Measurements and Cardiovascular Parameters

We observed that age, height, weight, BMI, waist circumference, and hip circumference did not significantly differ among the walking, swimming, and sedentary groups. However, the waist-to-hip ratio was notably higher in the sedentary group, suggesting a potentially higher risk of central obesity and associated cardiovascular complications. These findings align with previous research indicating that sedentary behavior may contribute to adverse changes in body composition and cardiovascular health [11,12].

Furthermore, while systolic and diastolic blood pressure did not exhibit significant differences among the groups, the lower pulse rate observed in the swimming group compared to both the walking and sedentary groups suggests potential cardiovascular benefits associated with regular swimming activity. Reduced pulse rate may indicate improved cardiovascular efficiency and autonomic modulation, reflecting a healthier cardiovascular profile among swimming participants. Swimmers may have slightly greater blood pressure due to the hemodynamic consequences of being in water, which might lead to increased peripheral vascular resistance. This suggests more research is needed on the long-term cardiovascular impacts of swimming in older adults [13].

Respiratory Endurance Parameters

The observed differences in respiratory endurance parameters, specifically the 40 mm Hg endurance test and maximum expiratory pressure, underscore the importance of regular physical activity, particularly aerobic exercises such as walking and swimming, in enhancing respiratory muscle strength and endurance among elderly individuals. These findings highlight the potential protective effects of physical activity against age-related declines in pulmonary function. Our results are consistent with previous studies by Battaglia et al., which demonstrated the positive impact of walking in natural environments, especially among geriatric populations, in delaying aging [14].

Moreover, our findings align with a meta-analysis conducted by Chen et al., which reported the beneficial effects of physical activity, particularly water-based exercises, on respiratory muscle strength and lung function [15]. The observed improvements in respiratory endurance parameters further emphasize the potential role of physical activity interventions in promoting respiratory health among aging populations [13].

Spirometry Parameters

Spirometry results revealed significant improvements in various respiratory parameters, including forced vital capacity (FVC), forced expiratory volume in the first second (FEV1), peak expiratory flow rate (PEFR), forced expiratory flow 25% (FEF-25), and maximum voluntary ventilation (MVV) in both the walking and swimming groups compared to the sedentary group. These findings suggest that engaging in regular walking or swimming activities may positively influence lung function and respiratory health among elderly individuals. The results are in line with the systematic review by Novotovo et al., showing that walking in the elderly population has been shown to improve respiratory parameters such as peak expiratory flow rate (PEFR), forced expiratory volume in 1 second (FEV1), and forced vital capacity (FVC) [16].

The significant improvements observed in FVC and FEV1 in the swimming group compared to the sedentary and walking groups highlight the potential superiority of swimming as an exercise modality for enhancing pulmonary function in older adults. The buoyancy and resistance provided by water during swimming may contribute to increased respiratory muscle strength and lung capacity, leading to more pronounced improvements in spirometry parameters [13].

These results indicate that both aerobic exercises like walking and non-weight-bearing activities like swimming can have a beneficial impact on lung function in older adults. The variations in respiratory parameters between the groups may be due to the varying demands on the respiratory system during different types of physical activity. This emphasizes the significance of including various exercise methods in physical activity programs to enhance respiratory health in older populations.

Limitations

The study has disadvantages such as a limited sample size, a sample from a limited cluster, and a cross-sectional methodology, which prevent making causal inferences and generalizing findings to larger groups. Future studies should use bigger sample sizes, longitudinal designs, and thorough assessments of participants' health status and lifestyle choices to better understand how various types of physical activity affect physiological outcomes in older populations.

Clinical implications and future directions

The study highlights the importance of promoting regular physical activity, particularly walking and swimming, to preserve respiratory health in aging populations. Healthcare professionals should encourage tailored exercise programs that focus on respiratory function. Future research should explore the long-term effects of different physical activities on respiratory health across diverse populations and investigate underlying mechanisms to inform targeted therapeutic strategies.

## Conclusions

Our study underscores the importance of regular physical activity in promoting respiratory health among elderly individuals. Engaging in activities like walking or swimming may confer significant benefits in terms of respiratory endurance and spirometry parameters, highlighting the potential role of exercise interventions in mitigating age-related declines in pulmonary function.
